# Changes in Racial Disparities in Mortality After Cancer Surgery in the US, 2007-2016

**DOI:** 10.1001/jamanetworkopen.2020.27415

**Published:** 2020-12-03

**Authors:** Miranda B. Lam, Katherine Raphael, Winta T. Mehtsun, Jessica Phelan, E. John Orav, Ashish K. Jha, Jose F. Figueroa

**Affiliations:** 1 Department of Health Policy and Management, Harvard T. H. Chan School of Public Health, Boston, Massachusetts; 2 Department of Radiation Oncology, Brigham and Women’s Hospital/Dana Farber Cancer Institute, Harvard Medical School, Boston, Massachusetts; 3 Department of Surgery, Brigham and Women’s Hospital/Dana Farber Cancer Institute, Harvard Medical School, Boston, Massachusetts; 4 Division of General Internal Medicine, Department of Medicine, Brigham and Women’s Hospital, Harvard Medical School, Boston, Massachusetts; 5 Department of Biostatistics, Harvard T. H. Chan School of Public Health, Boston, Massachusetts; 6 Department of Medicine, Brigham and Women’s Hospital, Harvard Medical School, Boston, Massachusetts

## Abstract

**Importance:**

Racial disparities are well documented in cancer care. Overall, in the US, Black patients historically have higher rates of mortality after surgery than White patients. However, it is unknown whether racial disparities in mortality after cancer surgery have changed over time.

**Objective:**

To examine whether and how disparities in mortality after cancer surgery have changed over 10 years for Black and White patients overall and for 9 specific cancers.

**Design, Setting, and Participants:**

In this cross-sectional study, national Medicare data were used to examine the 10-year (January 1, 2007, to November 30, 2016) changes in postoperative mortality rates in Black and White patients. Data analysis was performed from August 6 to December 31, 2019. Participants included fee-for-service beneficiaries enrolled in Medicare Part A who had a major surgical resection for 9 common types of cancer surgery: colorectal, bladder, esophageal, kidney, liver, ovarian, pancreatic, lung, or prostate cancer.

**Exposures:**

Cancer surgery among Black and White patients.

**Main Outcomes and Measures:**

Risk-adjusted 30-day, all-cause, postoperative mortality overall and for 9 specific types of cancer surgery.

**Results:**

A total of 870 929 cancer operations were performed during the 10-year study period. In the baseline year, a total of 103 446 patients had cancer operations (96 210 White patients and 7236 Black patients). Black patients were slightly younger (mean [SD] age, 73.0 [6.4] vs 74.5 [6.8] years), and there were fewer Black vs White men (3986 [55.1%] vs 55 527 [57.7%]). Overall national mortality rates following cancer surgery were lower for both Black (−0.12%; 95% CI, −0.17% to −0.06% per year) and White (−0.14%; 95% CI, −0.16% to −0.13% per year) patients. These reductions were predominantly attributable to within-hospital mortality improvements (Black patients: 0.10% annually; 95% CI, −0.15% to −0.05%; *P* < .001; White patients: 0.13%; 95% CI, −0.14% to −0.11%; *P* < .001) vs between-hospital mortality improvements. Across the 9 different cancer surgery procedures, there was no significant difference in mortality changes between Black and White patients during the period under study (eg, prostate cancer: 0.35; 95% CI, 0.02-0.68; lung cancer: 0.61; 95% CI, −0.21 to 1.44).

**Conclusions and Relevance:**

These findings offer mixed news for policy makers regarding possible reductions in racial disparities following cancer surgery. Although postoperative cancer surgery mortality rates improved for both Black and White patients, there did not appear to be any narrowing of the mortality gap between Black and White patients overall or across individual cancer surgery procedures.

## Key Points

**Question:**

What are the disparities in mortality after cancer surgery between Black and White patients from 2007 to 2016?

**Findings:**

In this cross-sectional study of 870 929 cancer operations over 10 years, overall mortality rates after cancer surgery decreased for both Black and White patients; however, no significant narrowing of the mortality gap between Black and White patients was observed. Mortality improvements were largely associated with within-hospital factors.

**Meaning:**

Overall, mortality rates following cancer surgery appear to be improving for both Black and White patients, but the gap in cancer surgery mortality rates between Black and White patients remains and does not appear to be narrowing.

## Teaser

This cross-sectional study examines changes in mortality rates over time in White and Black patients undergoing surgery for cancer.

## Introduction

Racial disparities in health care access, treatment, and outcomes, including surgery, in the US have been well documented. Black US residents have higher rates of mortality for most of the 15 leading causes of death in the US, including cancer, which is the second highest cause of death.^1^ In addition, Black patients face more challenges related to cancer care and treatment as they are more likely to receive their cancer diagnoses at more advanced stages than White patients^1^ and have higher rates of mortality following cancer surgery.^2,3,4,5,6^

In the past 15 years, there have been numerous efforts to improve oncologic surgical care, including the standards for optimal cancer care released by the American College of Surgeons’ Commission on Cancer,^7^ the efforts of the Alliance/American College of Surgeons Clinical Research Program to implement evidence-based practices in surgical oncology,^8^ and the Cancer Control Blueprint series, which identifies challenges in cancer care and sets goals for better care and outcomes.^9^ These initiatives have focused on overall improvements in cancer care but have given less attention to directly reducing disparities in cancer mortality by targeting populations with higher-than-average mortality rates, specifically Black patients. Prior studies, many of which preceded the implementation of these initiatives, showed increased mortality rates for Black patients following oncologic surgery compared with White patients, and these differences were not only a function of advanced stage or disease severity.^10^ There is evidence, for example, that racial disparities can be partially explained by Black patients receiving care at lower quality hospitals.^11^

More recent work showed a narrowing in the disparity gap between Black and White patients following non–cancer-related surgery, which was not due to shifting regionalization of care.^12^ However, it is not clear whether recent efforts to improve oncologic surgical are associated with disparities among patients undergoing cancer surgery over time. If quality-of-care improvements have been widespread, then hospitals may have elevated the standard of care for both Black and White patients, thereby reducing disparities. However, if institutions that serve mostly White patients improve while hospitals that serve mostly Black patients do not, wider disparities may result. Empirical data would be useful to address this issue.

Therefore, in this study, we sought to examine changes over 10 years—a period that would provide a long-term view of mortality changes. Although a broad range of services is necessary to treat patients with cancer, including screening and timely access to high-quality medical and radiation oncology care, we chose to focus on surgery to observe possible changes in the quality in surgical care and identify future efforts to improve care and narrow the mortality gap between Black and White patients with cancer. We had 3 specific questions. First, have disparities in cancer surgery mortality for Black and White patients changed over a 10-year period? Second, are disparities in cancer surgery mortality rates primarily due to changes in care within hospitals (by minimizing care discrepancies within institutions) or between hospitals (as a result of Black patients shifting to hospitals that provide higher quality care)? Third, do these trends differ by cancer type? We hypothesized that the mortality gap between Black and White patients with cancer narrowed during this time.

## Methods

### Data Sources

We linked several data sources for this study. The Medicare Provider Analysis and Review Files and Medicare Inpatient Claims data were linked to the Beneficiary Denominator File and Medicare Enrollment Database,^13^ which provided patient-level variables, including basic demographic characteristics, primary causes and dates of hospitalization, comorbidities, mortality, and procedures. We used the American Hospital Association annual survey and excluded federal hospitals and specialty hospitals (eg, psychiatric hospitals and children’s hospitals).^14^ Next, we used the Area Health Resource Files to obtain community-level variables, including demographic and socioeconomic data (eg, median household income [MHI] and average level of education) of the community in which the patient lived.^15^ We linked these sources to Medicare data using county codes. The study followed the Strengthening the Reporting of Observational Studies in Epidemiology (STROBE) reporting guideline for cross-sectional studies.^16^ The institutional review board at the Harvard T. H. Chan School of Public Health approved this study. The need for informed consent was waived because the data were deidentified.

### Patient Cohorts

Using claims data from January 1, 2007, to November 30, 2016, from the 100% Medicare Provider Analysis and Review (2007, 2008, and 2010) and Medicare (2009 and 2011-2016) inpatient files, we identified fee-for-service beneficiaries enrolled in Medicare Part A who had a major surgical resection for colorectal, bladder, esophageal, kidney, liver, ovarian, pancreatic, lung, or prostate cancer. We used diagnosis and procedure codes from *International Classification of Disease, Ninth Revision, Clinical Modification* (eTable 1 in the Supplement).^17^ We excluded patients who had undergone 2 or more different oncologic procedures on the same day and excluded hospitals that performed less than 25 procedures over the study period. All analyses were carried out from August 6 to December 31, 2019, using SAS, version 9.4 (SAS Institute Inc).

### Statistical Analysis

#### Outcomes

Our primary outcome of interest was risk-adjusted 30-day, all-cause, postoperative mortality. This outcome was defined as death within 30 days of the operation.^6^ We defined race according to the race variable in the Medicare enrollment denominator file. We excluded Hispanic patients and focused on the difference between non-Hispanic Black and non-Hispanic White patients because they are the 2 largest racial groups in the Medicare population, and the race variable has been widely tested and validated for these 2 groups.^18^ We defined disparity as the difference between the annual risk-adjusted postoperative mortality rates of Black vs White patients. Our main factors were calendar year, which was included as a continuous variable for the race of the patient (Black or White) and an interaction term for year and race. The interaction term determined whether mortality rates in Black and White patients were parallel, converging, or diverging over time. A 2-sided *P* < .05 was considered statistically significant.

#### Examining Racial Disparities in Mortality

For all types of cancer surgery, we first compared patient characteristics and comorbidities in the baseline year and ending year between Black and White patients. We then used a multivariable, patient-level linear probability regression model to examine the association between race and postoperative mortality over time. We chose a linear probability model to preserve the interpretability of linear trends in mortality rates. We used a generalized estimating equation approach to adjust for correlated outcomes within hospitals over time. We ran our main model for all types of cancer surgery together, then separately for each surgery. To ensure that changes in mortality rates were not due to changes in patient severity, all models controlled for age, sex, Medicaid eligibility, Elixhauser comorbidities, and measures of neighborhood socioeconomic status (MHI and proportion of adult patients who had completed high school). Patients without a valid county code were assigned the average value for that variable. This information allowed us to calculate overall and procedure-specific, risk-adjusted estimates in the average change in mortality over the study period for Black and White patients and assess whether the changes were consistent over time and across procedures.

#### Mechanism of Changes in Disparities Between vs Within Hospitals

To determine what proportion of mortality changes between Black and White patients was due to within-hospital parameters (ie, reductions in differential care within the same institution) vs between-hospital factors (ie, shifting of patients from low-quality to higher-quality hospitals), we ran patient-level linear probability regression models as described in the previous section with the addition of hospital fixed effects. The models with hospital fixed effects provided us with the within-hospital outcome, and the difference between the results from this fixed-effects model and those from the original model (without hospital fixed effects) provided us with the between-hospital changes. These models included the 4% of hospitals (122 of 2761) with 0% 30-day mortality, which accounted for 0.6% of operations (5249 of 870 929).

#### Sensitivity Analyses

We performed several sensitivity analyses. First, we reran our analyses using logistic regression. Although the linear probability model was chosen for interpretability, we wanted to confirm the results with logistic regression using random effects for hospitals to retain hospitals with 0% mortality (eTable 2 in the Supplement). Second, we reran our models based on MHI and high-school completion measured repeatedly over time (2007-2009, 2010-2012, 2013-2014, and 2015-2016) (eTable 3 in the Supplement). Third, we excluded MHI and high-school completion from our model (eTable 4 in the Supplement). Fourth, we included a measure of frailty/functional status in our model (eTable 5 in the Supplement).^19,20^ Fifth, although the primary model adjusted for cancer type, we reran our analyses to include adjustment for type and extent of surgery (eTable 1 and eTable 6 in the Supplement). Sixth, although our study was focused on postoperative mortality, we reran the linear regression model to determine surgical complication rates in our cohort (eTable 7 and eTable 8 in the Supplement).^21,22^

## Results

### Baseline Patient Characteristics

There were a total of 870 929 cancer operations in 2761 hospitals during the 10-year period of the study. In the baseline year, there were 96 210 White patients and 7236 Black patients; compared with White patients, Black patients were slightly younger (mean [SD], 73.0 [6.4] vs 74.5 [6.8] years). The cohort included 3986 Black (55.1%) vs 55 527 White (57.7%) men and 3250 Black (44.9%) vs 40 683 White (42.3%) women (Table 1). In addition, Black patients lived in counties with a lower MHI ($47 770 vs $50 343) and lower percentage of people with at least a high school diploma (85.5% vs 87.6%) (Table 1). In 2016, these patterns were consistent between Black and White patients.

**Table 1.** Baseline Patient Characteristics

Characteristic	Total, No. (%)			
	Year 2007 (n = 103 446)		Year 2016 (n = 71 766)	
	White (n = 96 210)	Black (n = 7236)	White (n = 66 204)	Black (n = 5562)
Age, mean (SD), y	74.5 (6.8)	73.0 (6.4)	74.9 (6.8)	73.1 (6.4)
Sex				
Men	55 527 (57.7)	3986 (55.1)	38 179 (57.7)	3156 (56.7)
Women	40 683 (42.3)	3250 (44.9)	28 025 (42.3)	2406 (43.3)
Medicaid eligibility	7310 (7.6)	1878 (26.0)	5229 (7.9)	1442 (25.9)
Congestive heart failure	7266 (7.6)	561 (7.8)	4400 (6.6)	453 (8.1)
Hypertension	48 726 (50.6)	4502 (62.2)	35 315 (53.3)	3505 (63.0)
Chronic lung disease	21 366 (22.2)	1226 (16.9)	12 174 (18.4)	812 (14.6)
Diabetes	15 312 (15.9)	1858 (25.7)	13 173 (19.9)	1659 (29.8)
Liver disease	1037 (1.1)	87 (1.2)	1553 (2.3)	155 (2.8)
Kidney failure	4997 (5.2)	718 (9.9)	4589 (6.9)	689 (12.4)
Obesity	3185 (3.3)	291 (4.0)	5749 (8.7)	609 (10.9)
Depression	3039 (3.2)	122 (1.7)	2890 (4.4)	122 (2.2)
County-level median household income, median (IQR), $	50 343 (15 266)	47 770 (13 328)	51 903 (16 732)	47 902 (14 385)
Obtained high school diploma, median (IQR)	87.6 (6.1)	85.5 (6.8)	87.6 (6.2)	85.5 (7.1)

Abbreviation: IQR, interquartile range.

### Overall Mortality Trends

Combining all types of cancer surgery, in the baseline years (2007-2008), Black patients had a composite mortality rate of 4.84%, compared with 4.29% for White patients, with a Black-White difference of 0.55% (95% CI, 0.21%-0.90%; *P* = .002) (Table 2). Composite mortality rates decreased significantly for both Black patients (−0.12%; 95% CI, −0.17% to −0.06% per year) and White patients (−0.14%; 95% CI, −0.16% to −0.13% per year). However, the racial difference did not significantly narrow or widen over the study period (0.03%; 95% CI, −0.03% to 0.08%; *P* = .36) (Figure; Table 2). In 2015-2016, Black patients had a composite mortality rate of 3.81% compared with White patients, who had a composite mortality of 3.09%, with a difference between the races of 0.73% (95% CI, 0.39%-1.06%; *P* < .001). This finding represents a significant difference in mortality between the baseline years and the final period of this study for both Black and White patients (0.73%; 95% CI, 0.39-1.06; *P* < .001).

**Table 2.** Risk-Adjusted 30-Day Postoperative Overall and Within-Hospital Mortality for Cancer Surgery

Variable	Mortality (2007-2008), %	Average annual change, % (95% CI)		Mortality (2015-2016), %
		Overall mortality	Within-hospital mortality^a^	
Black	4.84	−0.12 (−0.17 to −0.06)	−0.10 (−0.15 to −0.05)	3.81
White	4.29	−0.14 (−0.16 to −0.13)	−0.13 (−0.14 to −0.11)	3.09
Difference, % (95% CI)^b^	0.55 (0.21-0.90)	0.03 (−0.03 to 0.08)	0.03 (−0.02 to 0.08)	0.73 (0.39-1.06)
*P* value	.002	.36	.28	<.001

^a^ Between-hospital trends can be calculated as overall trends minus within-hospital trends.

^b^ Black mortality minus White mortality might not equal difference because of rounding.

**Figure.**
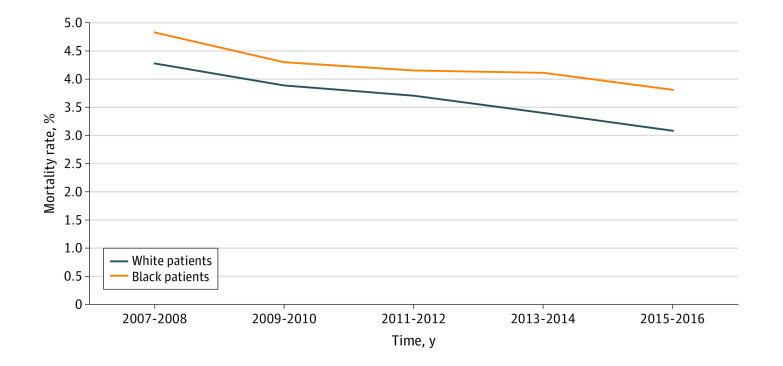
Risk-Adjusted 30-Day Postoperative Mortality for Cancer Operations

All sensitivity analyses (logistic regression, changing MHI and high-school completion over time, excluding MHI and high-school completion measured repeatedly over time, including frailty/functional status and type and extent of surgery) showed similar results to the main analysis (eTables 2-6 in the Supplement). We also found similar results using an alternative outcome measuring surgical quality (eTable 8 in the Supplement). Surgical complications decreased over time for both Black and White patients, but at equal rates, so that the complications for Black patients was significantly higher than for White patients, both in the baseline years and final period of the study.

### Between- vs Within-Hospital Mortality Trends

The within-hospital mortality rate for Black patients between 2007 to 2016 significantly decreased by 0.10% annually (95% CI, −0.15% to −0.05%; *P* < .001). Similarly, the within-hospital mortality rate for White patients significantly decreased by 0.13% annually (95% CI, −0.14% to −0.11%; *P* < .001). All of the 0.03% reduction in the composite Black mortality trend was associated within-hospital effects, that is, reduction in differential care within the same institution. None of the reduction in mortality was attributed to a between-hospital effect, that is, shifts in the number of Black patients from lower- to higher-quality hospitals (Table 2).

### Mortality Trends by Cancer Surgery

When we examined cancer surgery–specific mortality rates across the 9 different procedures, we found that mortality was decreasing for both Black and White patients between 2006 and 2017 for all cancer operations except for Black patients undergoing prostate cancer surgery (0.01% increase in average annual change in overall mortality) (Table 3). However, there were no significant differences in mortality changes between Black and White patients, as demonstrated by the nonsignificant difference between Black and White patients in the yearly mortality change. Therefore, it appears that the disparities are neither widening nor narrowing by cancer surgery examined. At baseline (2007-2008), Black patients had higher mortality rates than White patients for the following 6 cancer operations: prostate, esophagus, pancreas, lung, kidney, and colorectal. However, mortality was significantly higher for only prostate, lung, and kidney cancer surgery. In the final time period (2015-2016), Black patients continued to have higher mortality rates for all the types of surgery examined except esophageal cancer, and only Black patients undergoing colorectal cancer surgery had significantly higher mortality. For each cancer surgery, the mortality change appeared to be associated with within-hospital differences (ie, differential care within the same institution).

**Table 3.** Risk-Adjusted 30-Day Postoperative Overall and Within-Hospital Mortality by Cancer Surgery

Surgery type	Mortality (2007-2008), %	Average annual change, % (95% CI)		Mortality (2015-2016), %
		Overall mortality	Within-hospital mortality^a^	
**Prostate (n = 137 657)**				
Black	0.54	0.01	0.01	0.57
White	0.28	−0.01	−0.01	0.22
Difference, % (95% CI)^b^	0.26 (0.02-0.54)	0.02 (−0.03 to 0.07)	0.02 (−0.01 to 0.05)	0.35 (0.02-0.68)
**Bladder (n = 37 122)**				
Black	4.02	−0.22	−0.28	3.14
White	4.09	−0.10	−0.10	3.37
Difference, % (95% CI)^b^	−0.07 (−2.09 to 1.95)	−0.12 (−0.45 to 0.21)	−0.18 (−0.51 to 0.14)	−0.23 (−2.17 to 1.71)
**Esophagus (n = 7431)**				
Black	10.37	−0.90	−1.08	4.71
White	6.49	−0.11	0.02	6.10
Difference, % (95% CI)^b^	3.88 (−3.32 to 11.07)	−0.79 (−2.04 to 0.46)	−1.10 (−2.13 to −0.07)	−1.40 (−8.35 to 5.56)
**Pancreas (n = 25 126)**				
Black	6.71	−0.34	−0.21	4.04
White	5.15	−0.18	−0.11	3.71
Difference, % (95% CI)^b^	1.57 (−1.32 to 4.46)	−0.17 (−0.55 to 0.22)	−0.10 (−0.43 to 0.23)	0.33 (−1.68 to 2.34)
**Lung (n = 163 723)**				
Black	4.80	−0.24	−0.21	3.01
White	3.96	−0.19	−0.17	2.39
Difference, % (95% CI)^b^	0.84 (0.07-1.75)	−0.05 (−0.18 to 0.09)	−0.04 (−0.17 to 0.08)	0.61 (−0.21 to 1.44)
**Liver (n = 8551)**				
Black	5.23	−0.27	−0.42	4.64
White	6.89	−0.39	−0.39	3.89
Difference, % (95% CI)^b^	−1.65 (−5.55 to 2.25)	0.12 (−0.40 to 0.64)	−0.03 (−0.62 to 0.57)	0.75 (−2.07 to 3.57)
**Kidney (n = 104 789)**				
Black	2.89	−0.11	−0.10	1.75
White	1.97	−0.04	−0.03	1.56
Difference, % (95% CI)^b^	0.92 (0.15-1.68)	−0.07 (−0.18 to 0.04)	−0.07 (−0.17 to 0.03)	0.20 (−0.47 to 0.86)
**Colorectal (n = 348 662)**				
Black	6.83	−0.09	−0.07	5.82
White	6.34	−0.16	−0.14	4.95
Difference, % (95% CI)^b^	0.50 (−0.08 to 1.07)	0.07 (−0.03 to 0.17)	0.07 (−0.03 to 0.16)	0.87 (0.20-1.53)
**Ovarian (n = 37 868)**				
Black	4.65	−0.15	−0.11	4.75
White	5.49	−0.32	−0.27	2.91
Difference, % (95% CI)^b^	−0.83 (−2.82 to 1.15)	0.17 (−0.13 to 0.47)	0.15 (−0.15 to 0.45)	1.83 (−0.23 to 3.90)

^a^ Between-hospital trends can be calculated as overall trends minus within-hospital trends.

^b^ Black mortality minus White mortality might not equal difference because of rounding.

## Discussion

In this national study of Medicare beneficiaries, we found that although the gap in 30-day, all-cause, postoperative mortality was decreasing in both Black and White patients, the disparity gap between the racial groups persisted over time across a range of cancer surgery types. Lowered mortality rates for both groups were associated with within-hospital improvements, that is, improvements in outcomes within institutions, instead of from patients shifting from low- to high-quality hospitals. When the data were examined by cancer surgery, we saw decreases in mortality for both Black and White patients across 7 of the 9 types of cancer surgery. Taken together, these findings provide mixed news for policy makers interested in seeing reductions in disparities in mortality after cancer surgery given that we observed an overall decrease in mortality for both Black and White patients but no improvement in the disparity gap over time.

There are several reasons why the disparity gap continues to persist. First, we found higher rates of surgical complications in Black patients compared with White patients, which may explain some of the persistent disparity gap. In addition, Black patients have higher rates of developing and dying from invasive cancers compared with White patients,^23^ increased exposure to risk factors that place them under a disproportionate burden of disease,^24^ and greater likelihood of having the cancer diagnosed at a later stage^2,25^ and treated by physicians at lower-volume hospitals.^26^ Our work is consistent with other literature reporting that Black patients experienced higher rates of mortality after oncologic surgery than their White counterparts.^4,6,10,27,28,29,30^ Our work expands on this literature by looking at more contemporary data that cover the time period of recent surgical quality improvement efforts, and it also evaluates these changes across multiple types of cancer surgery.

Furthermore, there are potential explanations for why we observed overall improvements in mortality for cancer surgery but no improvements in racial disparities. During the period of our study, there were several policy changes incentivizing hospitals to focus on their quality improvement efforts, including value-based purchasing,^31^ hospital performance measures,^32^ and the accountable care organizations, which may have had some positive spillovers to surgery.^33^ It is also possible that there have been improvements in surgical practice^34^ and technologies (eg, robot-assisted cancer surgery, which may improve short-term survival for some cancers^35^), precipitating better cancer surgery outcomes across the board, but not narrowing disparities. Other efforts have focused on reducing procedures at low-volume surgery hospitals and preventing surgeons who perform relatively few operations from performing certain surgical procedures.^36^ Surgical checklists are also being implemented more widely and are associated with significant decreases in postoperative mortality.^37^

However, most recent efforts have not directly addressed the structural racism that may be underpinning the gap in outcomes. Race scholars have argued that racism produces significantly higher rates of morbidity and mortality, and decreased overall well-being.^38^ Racism exists in health institutions, including in oncologic care, and likely plays a role in perpetuating worse outcomes among Black patients with cancer.^39^ It is also possible that the disparity gap observed in cancer surgery may be due to upstream and/or downstream issues from the surgery (eg, late referrals, which may lead to late presentation at the time of surgery; failure to rescue; poor follow-up after discharge; and limited resources in the community), and that different policies and intervention may be needed to address disparities in cancer surgery.

Another possibility for the overall improvements in mortality is that the patients are undergoing surgery at a less-advanced stage of cancer. Improvements in screening and detection may mean that patients undergo surgery at earlier stages, which could result in lower mortality rates. However, Black patients are still diagnosed with cancer at later stages than their White counterparts.^25^ The results of previous studies on stage and perioperative mortality are mixed^40,41,42,43^ but could contribute to the disparity gap given our inability to adjust for cancer stage. Furthermore, the prevalence of most chronic diseases seems to be increasing among Black patients. Comorbidity status is related to the risk of perioperative mortality, which may explain some of the mortality gap. More work is needed to better understand why disparities are not lessening for cancer surgery and what steps can be taken to possibly eliminate the postoperative mortality gap.

### Limitations

This study has limitations. First, we used administrative claims data, which lack clinical data. Because we did not have information on cancer stage at diagnosis, we could not fully adjust for differences in risk between Black and White patients. However, by examining changes over time, we could compare the performance of hospitals over the 10-year period of study. Second, because we relied on Medicare claims data, we do not know whether our findings apply to the US population not covered by Medicare. It would be useful to see if these results are similar for patients younger than 65 years or are uninsured/underinsured. Third, our population of Black patients undergoing cancer surgery was much smaller than the population of White patients, which may have skewed mortality rates for individual cancers. In addition, although mortality and surgical complications are important outcomes, they reflect only 2 dimensions of measuring disparities. There are outcomes of surgical quality that are important to investigate further.

## Conclusions

This study noted decreased postoperative 30-day mortality associated with cancer surgery overall for both Black and White patients, yet we observed no closing of the disparity gap in cancer mortality over time. All improvements in mortality seem to be associated with within-hospital vs between-hospital differences. These findings suggest that although interventions, policies, and advancements in technology have improved mortality for all patients, they have not targeted disparities between Black and White patients. Understanding why overall cancer surgery mortality has decreased while the mortality gap has not closed may provide further insights into how to provide better care for all patients.

## Notes

Supplement. **eTable 1.** Cohort Selection **eTable 2.** Logistic Regression Analysis of Risk-Adjusted 30-Day Postoperative Overall and Within-Hospital Mortality Trends for Cancer Surgery **eTable 3.** Risk-Adjusted 30-Day Postoperative Overall and Within-Hospital Mortality for Cancer Surgery; County Level SES Variables (Educational Attainment and MHI) Changed Over Time **eTable 4.** Risk-Adjusted 30-Day Postoperative Overall and Within-Hospital Mortality for Cancer Surgery; Without County-Level SES Variables (Educational Attainment and MHI) **eTable 5.** Risk-Adjusted 30-Day Postoperative Overall and Within-Hospital Mortality for Cancer Surgery; Including Adjustment for Frailty/Functional Status **eTable 6.** Risk-Adjusted 30-Day Postoperative Overall and Within-Hospital Mortality for Cancer Surgery; Including Adjustment for Type and Extent of Surgery **eTable 7.** Surgical Complications **eTable 8.** Overall and Within-Hospital Complications for Cancer Surgery
