# Supplementary material for: Changes in Racial Disparities in Mortality After Cancer Surgery in the US, 2007-2016
Source: JAMA Netw Open. 2020 Dec 3;3(12):e2027415. doi: 10.1001/jamanetworkopen.2020.27415 (PMC7716190; doi:10.1001/jamanetworkopen.2020.27415)
Supplement: Supplement. — eTable 1. Cohort Selection eTable 2. Logistic Regression Analysis of Risk-Adjusted 30-Day Postoperative Overall and Within-Hospital Mortality Trends for Cancer Surgery eTable 3. Risk-Adjusted 30-Day Postoperative Overall and Within-Hospital Mortality for Cancer Surgery; County Level SES Variables (Educational Attainment and MHI) Changed Over Time eTable 4. Risk-Adjusted 30-Day Postoperative Overall and Within-Hospital Mortality for Cancer Surgery; Without County-Level SES Variables (Educational Attainment and MHI) eTable 5. Risk-Adjusted 30-Day Postoperative Overall and Within-Hospital Mortality for Cancer Surgery; Including Adjustment for Frailty/Functional Status eTable 6. Risk-Adjusted 30-Day Postoperative Overall and Within-Hospital Mortality for Cancer Surgery; Including Adjustment for Type and Extent of Surgery eTable 7. Surgical Complications eTable 8. Overall and Within-Hospital Complications for Cancer Surgery [file jamanetwopen-e2027415-s001.pdf]

Supplementary Online Content

Lam MB, Raphael K, Mehtsun WT, et al. Changes in racial disparities in mortality after cancer surgery in the US, 2007-2016. *JAMA Netw Open*. 2020;3(12):e2027415. doi:10.1001/jamanetworkopen.2020.27415

eTable 1. Cohort Selection

eTable 2. Logistic Regression Analysis of Risk-Adjusted 30-Day Postoperative Overall and Within-Hospital Mortality Trends for Cancer Surgery

eTable 3. Risk-Adjusted 30-Day Postoperative Overall and Within-Hospital Mortality for Cancer Surgery; County Level SES Variables (Educational Attainment and MHI) Changed Over Time

eTable 4. Risk-Adjusted 30-Day Postoperative Overall and Within-Hospital Mortality for Cancer Surgery; Without County-Level SES Variables (Educational Attainment and MHI)

eTable 5. Risk-Adjusted 30-Day Postoperative Overall and Within-Hospital Mortality for Cancer Surgery; Including Adjustment for Frailty/Functional Status

eTable 6. Risk-Adjusted 30-Day Postoperative Overall and Within-Hospital Mortality for Cancer Surgery; Including Adjustment for Type and Extent of Surgery

eTable 7. Surgical Complications

eTable 8. Overall and Within-Hospital Complications for Cancer Surgery

This supplementary material has been provided by the authors to give readers additional information about their work.

© 2020 Lam MB et al. *JAMA Network Open.*

eTable 1. Cohort Selection

Cancer type	Diagnosis codes (ICD-9)	Procedure codes (ICD-9)
Prostate	185	603: Suprapubic prostatectomy[1]<br>604: Retropubic prostatectomy[1]<br>605: Radical prostatectomy[1]<br>6061: Local excision of lesion of prostate[1]<br>6062: Perineal prostatectomy[1]<br>6069: Other prostatectomy[1]
Bladder	188 1880 1881 1882 1883 1884 1885 1886 1887 1888 1889	576: Partial cystectomy[2]<br>577: Total cystectomy[3]<br>5771: Radical cystectomy[3]<br>5779: Other total cystectomy[3]
Esophagus	150 1500 1501 1502 1503 1504 1505 1508 1509	424: Excision of esophagus[4]<br>4240: Esophagectomy, not otherwise specified[4]<br>4241: Partial esophagectomy[4]<br>4242: Total esophagectomy[5]<br>4399: Other total gastrectomy[5]
Pancreas	157 1570 1571 1572 1573 1574 1575 1576 1577 1578 1579	5251: Proximal pancreatectomy[6]<br>5252: Distal pancreatectomy[6]<br>5253: Radical subtotal pancreatectomy[6]<br>525: Partial Pancreatectomy[6]<br>5259: Other partial pancreatectomy[6]<br>5222: Other excision or destruction of lesion or tissue of pancreas or pancreatic duct[6]<br>526: Total pancreatectomy[7]<br>527: Radical pancreaticoduodenectomy[7]
Lung	162 1620 1621 1622 1623 1624 1625 1626 1627 1628 1629	322: Local Excision Or Destruction Of Lesion Or Tissue Of Lung[8]<br>329: Other excision of lung[8]<br>3229: Other local excision or destruction of lesion or tissue of lung[8]<br>3239: Other and unspecified segmental resection of lung[8]<br>3220: Thoracoscopic excision of lesion or tissue of lung[9]<br>3230: Thoracoscopic segmental resection of lung[9]<br>324: Lobectomy Of Lung[10]<br>3249: Other lobectomy of lung[10]<br>3241: Thoracoscopic lobectomy of lung[11]<br>3529: Other and unspecified pneumonectomy[12]<br>325: Complete Pneumonectomy[12]<br>3250: Thoracoscopic pneumonectomy[13]
Liver	155 1550 1551 1552	5022: Partial hepatectomy[14]<br>503: Lobectomy of liver[14]

© 2020 Lam MB et al. *JAMA Network Open.*

		502: Local Excision Or Destruction Of Liver Tissue Or Lesion[14]<br>5029: Other destruction of lesion of liver[14]
		504: Total hepatectomy[15]
		5059: Other transplant of liver[16]
Kidney	189 1890 18900 18901 1898 1891 23691	554: Partial nephrectomy[17]<br>5532: Open ablation of renal lesion or tissue[17]<br>5551: Nephroureterectomy[18]<br>5552: Nephrectomy of remaining kidney[18]<br>555: Complete Nephrectomy[18]<br>5554: Bilateral nephrectomy[18]
		5534: Laparoscopic ablation of renal lesion or tissue[19]
Colorectal	1530 1531 1532 1533 1534 1535 1536 1537 1538 1539 1540 1541	457: Open And Other Partial Excision Of Large Intestine[20]<br>4571: Open and other multiple segmental resection of large intestine[20]<br>4572: Open and other cecectomy[20]<br>4573: Open and other right hemicolectomy[20]<br>4574: Open and other resection of transverse colon[20]<br>4575: Open and other left hemicolectomy[20]<br>4576: Open and other sigmoidectomy[20]<br>4579: Other and unspecified partial excision of large intestine[20]
		458: Total Intra-Abdominal Colectomy[21]<br>4582: Open total intra-abdominal colectomy[21]<br>4583: Other and unspecified total intra-abdominal colectomy[21]
		4859: Other abdominoperineal resection of the rectum[22]<br>4861: Transsacral rectosigmoidectomy[22]<br>4862: Anterior resection of rectum with synchronous colostomy[22]<br>4863: Other anterior resection of rectum[22]<br>4864: Posterior resection of rectum[22]<br>4865: Duhamel resection of rectum[22]<br>4869: Other resection of rectum[22]<br>4840: Pull-through resection of rectum, not otherwise specified[22]<br>486: Other Resection Of Rectum[22]<br>484: Pull-Through Resection Of Rectum[22]<br>4841: Soave submucosal resection of rectum[22]<br>4852: Open abdominoperineal resection of the rectum[22]<br>4843: Open pull-through resection of rectum[22]<br>4849: Other pull-through resection of rectum[22]<br>4850: Abdominoperineal resection of the rectum, not otherwise specified[22]<br>485: Abdominoperineal Resection Of Rectum[22]

© 2020 Lam MB et al. *JAMA Network Open.*

		4581: Laparoscopic total intra-abdominal colectomy[23]<br>1732: Laparoscopic cecectomy[23]<br>1733: Laparoscopic right hemicolectomy[23]<br>1734: Laparoscopic resection of transverse colon[23]<br>1735: Laparoscopic left hemicolectomy[23]<br>1736: Laparoscopic sigmoidectomy[23]<br>1739: Other laparoscopic partial excision of large intestine[23]<br>4842: Laparoscopic pull-through resection of rectum[24]<br>4851: Laparoscopic abdominoperineal resection of the rectum[24]
Ovarian	1830	652: Local Excision Or Destruction Of Ovarian Lesion Or Tissue[25]<br>6539: Other unilateral oophorectomy[25]<br>6551: Other removal of both ovaries at same operative episode[25]<br>6552: Other removal of remaining ovary[25]<br>6549: Other unilateral salpingo-oophorectomy[25]<br>6561: Other removal of both ovaries and tubes at same operative episode[25]<br>6562: Other removal of remaining ovary and tube[25]<br>664: Total unilateral salpingectomy[25]<br>665: Total Bilateral Salpingectomy[25]<br>654: Unilateral Salpingo-Oophorectomy[25]<br>656: Bilateral Salpingo-Oophorectomy[25]<br>541: Laparotomy[25]<br>5411: Exploratory laparotomy[25]<br>544: Excision or destruction of peritoneal tissue[25]<br>6531: Laparoscopic unilateral oophorectomy[26]<br>6553: Laparoscopic removal of both ovaries at same operative episode[26]<br>6554: Laparoscopic removal of remaining ovary[26]<br>6541: Laparoscopic unilateral salpingo-oophorectomy[26]<br>6563: Laparoscopic removal of both ovaries and tubes at same operative episode[26]<br>6564: Laparoscopic removal of remaining ovary and tube[26]<br>6831: Laparoscopic supracervical hysterectomy [LSH][27]<br>6841: Laparoscopic total abdominal hysterectomy[27]<br>6851: Laparoscopically assisted vaginal hysterectomy (LAVH)[27]<br>6861: Laparoscopic radical abdominal hysterectomy[27]<br>6871: Laparoscopic radical vaginal hysterectomy [LRVH][27]<br>5421: Laparoscopy[27]<br>688: Pelvic evisceration[28]<br>683: Subtotal Abdominal Hysterectomy[28]<br>6839: Other and unspecified subtotal abdominal hysterectomy[28]

© 2020 Lam MB et al. *JAMA Network Open.*

		684: Total Abdominal Hysterectomy[28]<br>6849: Other and unspecified total abdominal hysterectomy[28]<br>685: Vaginal Hysterectomy[28]<br>6859: Other and unspecified vaginal hysterectomy[28]<br>686: Radical Abdominal Hysterectomy[28]<br>6869: Other and unspecified radical abdominal hysterectomy[28]<br>687: Radical Vaginal Hysterectomy[28]<br>6879: Other and unspecified radical vaginal hysterectomy[28]<br>689: Other and unspecified hysterectomy[28]

[1-28] Sensitivity analysis (eTable 6) includes adjustment for type and extent of surgical operation based on these 28 groups

© 2020 Lam MB et al. *JAMA Network Open.*

eTable 2. Logistic Regression Analysis of Risk-Adjusted 30-Day Postoperative Overall and Within-Hospital Mortality Trends for Cancer Surgery

	Overall Mortality[1] (2007-2008)	Yearly Odds Ratio (95% Confidence Interval)		Overall Mortality (2015-2016)
		Overall Mortality	Within-Hospital Mortality	
**Black**	4.84%	0.97 (0.95-0.98)	0.97 (0.96-0.99)	3.81%
**White**	4.29%	0.96 (0.95-0.96)	0.96 (0.96-0.97)	3.09%
**Odds Ratios (95%CI) for Mortality, Blacks vs Whites**	1.14 (1.02-1.26) p=0.016	1.01 (0.99-1.03) p=0.22	1.01 (0.99-1.03) p=0.20	1.24 (1.09-1.41) p=0.001

[1]Overall mortality rates are from the linear probability model while the odds ratios, confidence interval and p-values are from the analogous logistic regression model.

© 2020 Lam MB et al. *JAMA Network Open.*

eTable 3. Risk-Adjusted 30-Day Postoperative Overall and Within-Hospital Mortality for Cancer Surgery; County Level SES Variables (Educational Attainment and MHI) Changed Over Time[1]

	Overall Mortality (2007-08)	Average Annual Change (95% Confidence Interval)		Overall Mortality (2015-16)
		Overall Mortality	Within-Hospital Mortality	
**Black**	4.79%	-0.10% (-0.15%, -0.05%)	-0.10% (-0.15%, -0.05%)	3.91%
**White**	4.24%	-0.13% (-0.14%, -0.11%)	-0.13% (-0.14%, -0.11%)	3.19%
**Difference**	0.56% (0.21%, 0.90%) p=0.002	0.02% (-0.03, 0.08%) p=0.38	0.03% (-0.02, 0.08%) p=0.28	0.72% (0.39%, 1.06%) p<0.001

[1] 2007-2009, 2010-2012, 2013-2014, 2015-16

© 2020 Lam MB et al. *JAMA Network Open.*

eTable 4. Risk-Adjusted 30-Day Postoperative Overall and Within-Hospital Mortality for Cancer Surgery; Without County-Level SES variables (Educational Attainment and MHI)

	Overall Mortality (2007-08)	Average Annual Change (95% Confidence Interval)		Overall Mortality (2015-16)
		Overall Mortality	Within-Hospital Mortality	
**Black**	4.89%	-0.12% (-0.17%, -0.07%)	-0.10% (-0.15%, -0.05%)	3.85%
**White**	4.29%	-0.14% (-0.16%, -0.13%)	-0.13% (-0.14%, -0.11%)	3.08%
**Difference**	0.60% (0.26%, 0.95%) p=0.006	0.03% (-0.03, 0.08%) p=0.36	0.03% (-0.02, 0.08%) p=0.28	0.78% (0.44%, 1.12%) p<0.001

© 2020 Lam MB et al. *JAMA Network Open.*

eTable 5. Risk-Adjusted 30-Day Postoperative Overall and Within-Hospital Mortality for Cancer Surgery; Including Adjustment for Frailty/Functional Status[1]

	Overall Mortality (2010)	Average Annual Change (95% Confidence Interval)		Overall Mortality (2016)
		Overall Mortality	Within-Hospital Mortality	
**Black**	5.89%	-0.24%	-0.18%	3.57%
**White**	3.41%	-0.13%	-0.11%	3.04%
**Difference**	2.48% (0.07%, 4.90%) p=0.0438	-0.11% (-0.31%, 0.10%) p=0.3100	-0.07% (-0.26%, 0.11%) p=0.4319	0.52% (0.03%, 1.02%) p=0.0393

[1]In order to adjust for frailty, we could only use a 5% sample in years 2010-2011, a 20% sample in 2012-2013, and a 100% sample in 2014-2016.

© 2020 Lam MB et al. *JAMA Network Open.*

eTable 6. Risk-Adjusted 30-Day Postoperative Overall and Within-Hospital Mortality for Cancer Surgery; Including Adjustment for Type and Extent of Surgery

	Overall Mortality (2007-08)	Average Annual Change (95% Confidence Interval)		Overall Mortality (2015-16)
		Overall Mortality	Within-Hospital Mortality	
**Black**	4.42%	-0.06%	-0.05%	3.92%
**White**	3.96%	-0.09%	-0.08%	3.23%
**Difference**	0.46% (0.12%, 0.81%) p=0.009	0.03% (-0.02%, 0.08%) p=0.289	0.03% (-0.02%, 0.09%) p=0.211	0.69% (0.35%, 1.03%) p<0.001

© 2020 Lam MB et al. *JAMA Network Open.*

eTable 7. Surgical Complications

Complication	Codes
Hemorrhage	Intraoperative hemorrhage 998.11<br>Postoperative hematoma 998.12
Anastomotic complication	Leak 998.6<br>Percutaneous abdominal drainage 54.91
Wound complication	Infection 998.5, 998.51, 998.59<br>Seroma 998.13<br>Dehiscence 998.3
Pulmonary complication	Respiratory tract complications 997.3<br>Acute bacterial pneumonia 481, 482.0-482.9, 485, 486<br>Acute respiratory failure 518.81<br>Tracheotomy 31.1, 31.29
Cardiac complication	Complications 997.1<br>Acute myocardial infarction 410.0-410.9
Neurological complication	Central nervous system complications 997.01-997.03<br>Acute cerebrovascular accident 431.00-431.91, 433.00-433.91, 434.00-434.91, 436, 437.1
Genitourinary tract complication	Urinary tract complications 997.5<br>Acute renal failure 584.1-584.9<br>Acute dialysis 38.95<br>Insertion of short-term dialysis catheter 39.95
Thromboembolic complication	Acute pulmonary embolism 415.1, 415.11, 415.19<br>Acute deep venous thrombosis 453.8, 453.9
Shock	Postoperative 998.0
Unexpected reoperations for surgical complications	Wound dehiscence 54.61<br>Removal of foreign body 54.92<br>Laparotomy 54.12

© 2020 Lam MB et al. *JAMA Network Open.*

eTable 8. Overall and Within-Hospital Complications for Cancer Surgery

	Overall Complications (2007-08)	Average Annual Change (95% Confidence Interval)		Overall Complications (2015-16)
		Overall Complications	Within-Hospital Complications[1]	
**Black**	27.09%	-0.37%	-0.32%	24.45%
**White**	23.24%	-0. 41%	-0.37%	19.98%
**Difference**	3.85% (3.85%, 4.55%) p<0.001	0.04% (-0.08%, 0.15%) p=0.53	0.05% (-0.06, 0.16%) p=0.36	4.48% (3.69%, 5.27%) p<0.001

[1]Between-hospital trends can be calculated as overall trends minus within-hospital trends

© 2020 Lam MB et al. *JAMA Network Open.*
